# Pharmacokinetics, metabolite profiling, safety and tolerability of YZJ-4729 tartrate, a novel G protein-biased μ-opioid receptor agonist, in healthy Chinese subjects

**DOI:** 10.3389/fphar.2023.1295319

**Published:** 2024-01-09

**Authors:** Yufeng Ni, Huaye Gao, Wen Ouyang, Guoping Yang, Minlu Cheng, Li Ding

**Affiliations:** ^1^ Department of Pharmaceutical Analysis, China Pharmaceutical University, Nanjing, China; ^2^ Yangtze River Pharmaceutical Group Co, Ltd., Taizhou, China; ^3^ Nanjing Jiening Pharmaceutical Technology Co, Ltd., Nanjing, China; ^4^ Department of Anesthesiology, The Third Xiangya Hospital of Central South University, Changsha, China; ^5^ Clinical Trial Research Center, The Third Xiangya Hospital of Central South University, Changsha, China; ^6^ Nanjing Clinical Tech. Laboratories Inc., Nanjing, China

**Keywords:** YZJ-4729, acute pain, G protein-biased μ-opioid receptor agonist, pharmacokinetics, metabolite profiling, safety

## Abstract

**Objective:** YZJ-4729 is a novel G protein-biased μ-opioid receptor agonist for the treatment of acute pain in adult patients who require intravenous opioid analgesic therapy. The aim of this study was to assess the pharmacokinetics, metabolite profiling, safety and tolerability of YZJ-4729 in healthy Chinese subjects following the single intravenous doses ranged from 0.2 mg to 6 mg.

**Methods:** This single-center, randomized, double-blind, placebo-controlled clinical study was conducted in 54 healthy male and female Chinese subjects after single ascending doses of YZJ-4729 tartrate (0.2, 0.5, 1.5, 3, 4.5, and 6 mg). Subjects in each cohort were assigned randomly to receive a single intravenous dose of YZJ-4729 tartrate injection or placebo at a ratio of 4:1. Pharmacokinetic characteristics, metabolite profiling, safety and tolerability profiles of the study drug were evaluated.

**Results:** Overall, YZJ-4729 was safe and well tolerated in healthy Chinese subjects. The study drug reached peak plasma concentrations nearly at the end of the infusion. After administration, YZJ-4729 was eliminated rapidly with a terminal elimination half-life of 0.862–2.50 h, and excreted little in human excreta. The maximum drug concentration and area under the plasma concentration-time curve increased with dose escalation across the entire dose range. YZJ-4729 experienced extensive metabolism in human body. A total of 19 metabolites were identified and the characteristic metabolic pathways involved hydroxylation, ketone formation, N-dealkylation and glucuronide conjugation. Metabolite M10 was the most abundant circulating metabolite, and represented over 10% of total drug-related systemic exposure. Further PK and safety evaluation of M10 was necessary.

**Conclusion:** The clinical study results laid a foundation for the further clinical studies of YZJ-4729 in patients.

**Clinical Trial Registration:** http://www.chinadrugtrials.org.cn, identifier CTR20222574.

## 1 Introduction

Pain is defined as “an unpleasant sensory and emotional experience associated with actual or potential tissue damage, or described in terms of such damage” by the International Association for the Study of Pain (IASP) (Raja et al., 2020). It is frequently categorized as acute or chronic (Phillips and Clauw, 2013). Acute pain is typically associated with surgical trauma and tissue injury (Lee and Neumeister, 2020). It usually lasts less than 1 month (Carr and Goudas, 1999). While, chronic pain is typically defined as persistent or recurrent pain (Raffaeli et al., 2021). It usually lasts longer than 3 months (Treede et al., 2015). Post-operative pain is an acute pain that immediately occurs after the surgical procedures (with a duration of no more than 7 days) (Gupta et al., 2010; Small and Laycock, 2020). Post-operative pain is mostly nociceptive (Vadivelu et al., 2010). It is regarded as the most common form of acute pain, which requires immediate management in clinic (Mitra et al., 2018; Ye et al., 2023). Poorly managed post-operative pain could lead to complications and prolonged rehabilitation (Ottoboni et al., 2019; Stuhlreyer and Klinger, 2022). Uncontrolled acute pain may develop into chronic pain and decrease the patients’ quality of life (Kehlet et al., 2006). The appropriate management of acute post-operative pain could help patients to shorten hospital stay, reduce medical cost and accelerate the recovery of physical function (Garimella and Cellini, 2013).

Opioid analgesics are the mainstay of therapy in the post-operative period (Rawal, 2016; Fu et al., 2023). They exhibit consistent and strong analgesia effect through the interaction with opioid receptors (Pathan and Williams, 2012). Opioid receptors belong to G protein-coupled receptors (GPCRs) (Wacher et al., 2017). They include three main subtypes referred to as the μ-, δ-, and ҡ-opioid receptors (MOR, DOR, and KOR, respectively), together with the nonclassical nociceptin opioid receptor (NOP) (Che et al., 2021). The activation of the MOR triggers downstream signaling through the heterotrimeric G proteins, resulting in analgesia, sedation, euphoria and physical dependence (Huang et al., 2015). Meanwhile, the activated MOR can also signal through β-arrestin-2, leading to the adverse effects of opioid analgesics including tolerance, respiratory depression, and constipation (Al-Hasani and Bruchas, 2011; Liu et al., 2023). Therefore, designing a novel MOR agonist, which elicits G protein activation with minimal β-arrestin-2 signaling, is a new research direction. Oliceridine, the first G protein-biased MOR agonist, has been approved by the U.S. Food and Drug Administration (FDA) in August 2020 (Faouzi et al., 2020). It is indicated in adults for the management of acute pain severe enough to require an intravenous (IV) opioid and for whom alternative treatments are inadequate (Markham, 2020). However, considering certain adverse reactions, such as respiratory depression and prolongation of the QT interval, there is still room for further improvement (Miyano et al., 2020).

**FIGURE 1:**
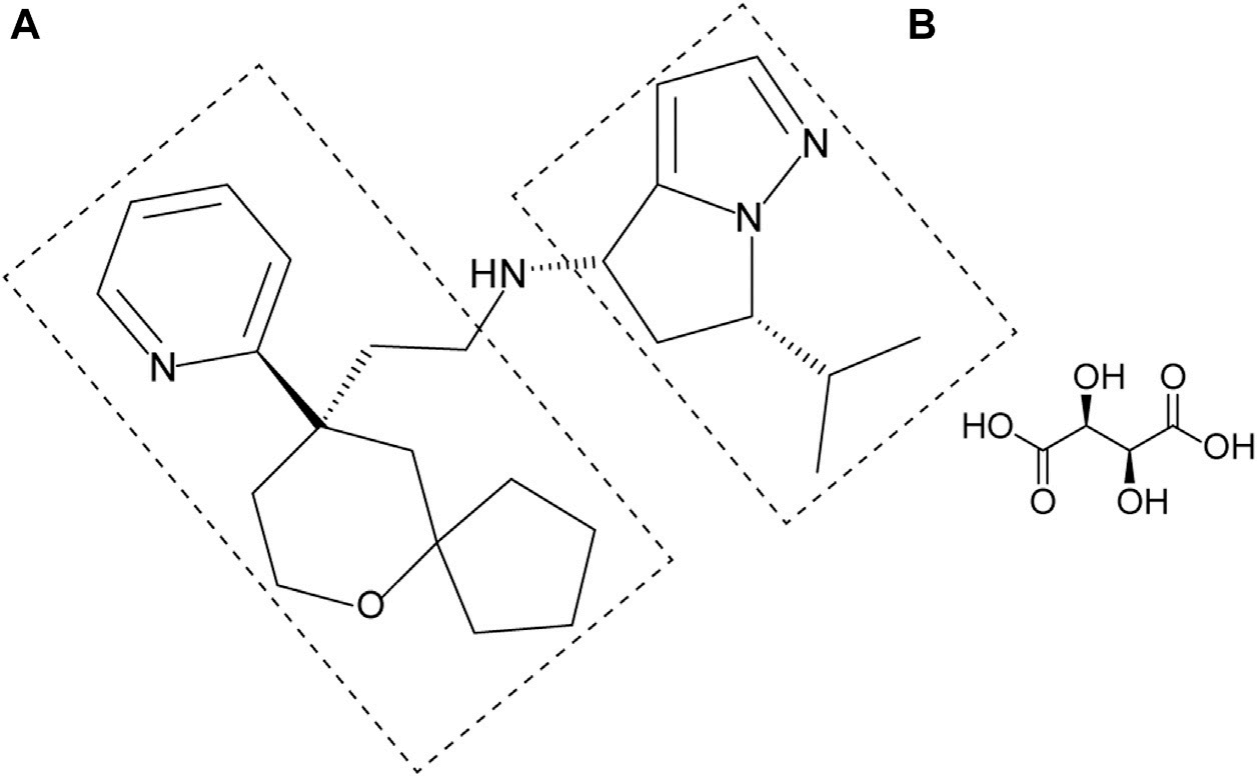
Chemical structure of YZJ-4729 tartrate.

YZJ-4729 tartrate, chemically known as (4S, 6S)-6-isopropyl-N-(2-((R)-9-(pyridine-2-yl)-6-oxaspiro[4,5]decan-9-yl)ethyl)-5,6-dihydro-4H-pyrrolo[1,2-b]pyrazol-4-amine L-tartrate (Figure 1), is a novel G protein-biased μ-opioid receptor agonist for the treatment of acute pain in adult patients who require intravenous opioid analgesic therapy. Its preparation method and medical application have been patented in China (Patent No. CN202110062634.X). Currently, YZJ-4729 is under Phase Ⅱ clinical trial. The pharmacological mechanism and activity of YZJ-4729 are similar to oliceridine.

YZJ-4729 exhibits its biased partial agonistic selective activity on MOR with a half-maximal effective concentration (EC_50_) of 0.017 μM. It also demonstrates certain antagonistic effects on KOR and DOR, with half-maximal inhibitory concentrations (IC_50_) of 1.100 μM and 6.962 μM, respectively. In the *in vitro* pharmacodynamic studies (unpublished data), compared with morphine, the relative maximal inhibitory effects of YZJ-4729 and oliceridine on forskolin-stimulated cAMP accumulation were 85.4% ± 4.8% and 91.4% ± 1.2%, respectively. This result indicated that YZJ-4729 could effectively activate MOR, and its pharmacological effect might be comparable to oliceridine. Moreover, when morphine was used as a reference, the maximum recruitments of β-arrestin-2 for YZJ-4729 and oliceridine were 4.2% ± 1.4% and 12.8% ± 0.9%, respectively. The result showed that YZJ-4729 had minimal recruitment of β-arrestin-2. The pharmacological profiling suggested a stronger G protein bias of YZJ-4729 than oliceridine in our test systems.

In the mouse hot-plate test, following subcutaneous administration of YZJ-4729 at doses of 1.5, 3, and 6 mg/kg, significant and dose-dependent analgesic effects were observed at 15, 30, 60, and 90 min post-dosing. The effective dose and median effective dose (ED_50_) were both 1.5 mg/kg. In the rat tail-flick test, after subcutaneous injection of YZJ-4729 at doses of 0.15, 0.3, 1, and 3 mg/kg, YZJ-4729 exhibited significant and dose-dependent analgesic effects at 15, 30, 60, and 90 min post-dosing. The effective dose was 0.15 mg/kg, and the ED_50_ was 0.3 mg/kg. In the rat hot-plate test, following subcutaneous administration of YZJ-4729 at doses of 0.15, 0.3, 1, and 3 mg/kg, significant and dose-dependent analgesic effects were observed at 15, 30, 60, and 90 min post-dosing. The effective dose was 0.15 mg/kg, and the ED_50_ was 0.3 mg/kg. In the rat arterial blood gas analysis model, the dosage was determined based on the results of the rat efficacy studies. In the rat tail-flick test and hot-plate test, after 15 min of administration, YZJ-4729 at 0.3 mg/kg showed comparable analgesic effects to morphine at 4.5 mg/kg and oliceridine at 0.6 mg/kg. Therefore, in this model, four times the 15-min equipotent analgesic effective dose was used to evaluate respiratory depression. After administration, the increase in blood carbon dioxide partial pressure (PCO_2_) and inhibitory effect on blood oxygen partial pressure (PO_2_) caused by YZJ-4729 were not significantly different from the control group. There was no reduction in blood pH value. The respiratory depression effects of YZJ-4729 were milder than morphine and oliceridine. These preclinical results suggest that YZJ-4729 may be a safer and more tolerable candidate in this medical field.

In the preclinical pharmacokinetic studies, after intravenous administration in rats and cynomolgus monkeys, YZJ-4729 underwent rapid metabolism, with the half-life values ranging from 0.171 to 0.342 h and 0.783–5.06 h, respectively. The plasma clearance (CL) was 75.0–93.9 mL/min/kg for rats and 22.8–27.2 mL/min/kg for cynomolgus monkeys. YZJ-4729 exhibited widespread tissue distribution (steady-state apparent volume of distribution, Vd_ss_, 1.13–1.28 L/kg in rats and 1.26–2.63 L/kg in cynomolgus monkeys). Across the entire studied dose range, the systemic exposure to YZJ-4729 in rats and cynomolgus monkeys increased proportionally with dose escalation. No gender difference was observed. These preclinical pharmacokinetic data laid a foundation on the subsequent clinical pharmacokinetic study.

Clinical pharmacokinetic (PK) study aims to describe the dynamic processes of drug absorption, distribution, metabolism and excretion (ADME) in the human body. The PK information provides the basis for establishing a safe and effective therapeutic dose schedule in clinical practice. Moreover, metabolite profiling aims to identify the composition of the metabolites and quantify their relative abundance. It is crucial for evaluating the safety profile of the study drug. If a human metabolite exceeds 10 percent of total drug-related exposure, it can raise a safety concern, and the nonclinical pharmacodynamic and toxicity studies of the metabolite should be conducted. The U.S. Food and Drug Administration (FDA) recommends that metabolite profiling should be conducted as early as feasible. Therefore, we conducted this study to evaluate the pharmacokinetics, metabolite profiling, safety and tolerability of YZJ-4729 in healthy Chinese subjects following the single intravenous administration.

## 2 Materials and methods

### 2.1 Drugs and reagents

YZJ-4729 tartrate injection, placebo and the reference standard were provided by Shanghai Haiyan Pharmaceutical Technology Co., Ltd. (Shanghai, China). LC-grade acetonitrile and methanol were purchased from Merck KGaA (Darmstadt, Germany). Ammonium acetate (NH_4_Ac) and formic acid (FA) of ACS-grade were obtained from Sigma-Aldrich (St. Louis, MO, United States). Ultrapure water was generated by a Milli-QTM system (Millipore, Bedford, MA, United States).

### 2.2 Study population

Eligible subjects were healthy male or female (non-lactating and non-pregnant) subjects, 18–45 years old with a body mass index (BMI) of 19.0–26.0 kg/m^2^. For the verification of the non-pregnant status in the premenopausal female subjects, before inclusion, they must have a negative result of the serum pregnancy test during screening, and on Day −1 visit, the result of the serum pregnancy test must also be negative. From signing the informed consent form (ICF) until 6 months after the last administration of the investigational drug, the female subjects must agree to one of the following measures to prevent pregnancy. 1) Complete abstinence (Periodic abstinence was not allowed). 2) From the screening day until 6 months after the last administration of the investigational drug, apart from the correct use of male condoms by their male partners, the female subjects must consistently use one of the listed contraceptive methods: a. intrauterine device (IUD) with an annual failure rate of <1%; b. female barrier method (cervical cap or uterine cap with spermicide); c. tubal ligation; d. vasectomy for their male partners; e. hormonal contraceptives; f. levonorgestrel implant; g. depot-medroxyprogesterone acetate injection; h. oral contraceptives (combination or progestin-only); i. vaginal contraceptive ring; j. transdermal contraceptive patch. Subjects were assessed as healthy based on a thorough evaluation by investigators, including medical history, physical examination, vital signs, 12-lead electrocardiogram (ECG), clinical laboratory tests, blood oxygen saturation assessments, abdominal ultrasound and chest X-ray. Meanwhile, on the day of admission, urine drug screening and alcohol breath testing were performed on the subjects. The urine drug screening included morphine, methamphetamine, ketamine, 3,4-methylenedioxy-methamphetamine (MDMA), and tetrahydrocannabinol (THC). Subjects were excluded if they met the exclusion criteria defined in the protocol. All subjects signed the ICF before screening.

### 2.3 Study design

This was a single-center, randomized, double-blind, placebo-controlled and single ascending dose study to evaluate the pharmacokinetics, metabolite profiling, safety and tolerability of YZJ-4729 in healthy Chinese subjects after intravenous administration. This dose-escalation study consisted of 6 dose cohorts (i.e., 0.2, 0.5, 1.5, 3, 4.5, and 6 mg). Dose escalation was only allowed when the previous dose was safe and well-tolerated in the subjects. Four subjects were enrolled in the starting dose cohort (0.2 mg), and all of them were administered the study drug. After that, ten subjects in each cohort randomly received YZJ-4729 tartrate injection or placebo at a ratio of 4:1. The randomization list was generated by using SAS statistical package version 9.4 (SAS Research and Development Co., Ltd., Chapel Hill, NC, United States). The randomization assignment was not revealed to subjects and personnel (including investigators, drug administrators, the sponsor and statisticians). The study drug and placebo were identical in appearance, weight, label and packaging to ensure the implementation of blinding. In this study, all subjects fasted overnight for at least 10 h before dosing and received single dose intravenous administration of YZJ-4729 tartrate injection or placebo over 30 min (±5 min) on Day 1. No water was allowed within 2 h before and after dosing. No food was permitted until 4 h after dosing. This study was conducted in compliance with the principles of the Declaration of Helsinki and the International Conference on Harmonization Good Clinical Practice guidelines. The study protocol and ICF were approved by the Third Xiangya Hospital Ethics Committee (Changsha, China). The study was registered at http://www.chinadrugtrials.org.cn with the identifier CTR20222574.

### 2.4 Sample collection

In all dose cohorts, the blood samples were taken for the pharmacokinetic assessment of YZJ-4729. Moreover, in the 1.5 mg dose cohort, in addition to the blood samples, the urine and feces samples were also collected. The biological samples in this dose cohort were used for the PK evaluation and metabolite profiling of the study drug. The blood samples were collected pre-dose and at 10, 20, 30 (just at the end of the infusion), 32, 35, 45 min, and 1, 1.5, 2, 2.5, 3.5, 4.5, 6.5, 8.5, 12.5 and 24.5 h after the start of the infusion. Plasma was separated and stored at −60 ∼ −90 °C until analysis. The urine samples were collected within 8 h pre-dose and the following time intervals: 0–4, 4–8, 8–12, 12–24, 24–48 and 48–72 h after the start of the infusion. The total volume of the urine samples in each collection period was recorded. The feces samples were collected within 24 h pre-dose and 72 h after the start of the infusion. The total weight of the feces samples was recorded. The urine and feces samples were stored at −60 ∼ −90 °C until analysis.

### 2.5 Pharmacokinetic assessments

Determination of YZJ-4729 in human plasma, urine and feces was conducted by using high performance liquid chromatography tandem mass spectrometry method (HPLC-MS/MS) with multiple reaction monitoring (MRM) and electrospray ionization in the positive mode (ESI^+^). The chromatographic separation was achieved by a Shimadzu LC-20ADXR HPLC system (Shimadzu, Kyoto, Japan). The MS detection was performed with API 4000 (Applied Biosystems/Sciex, Foster, United States) for plasma and feces, and 4000 QTRAP (Applied Biosystems/Sciex, Foster, United States) for urine, respectively. These methods were fully validated. The calibration ranges of YZJ-4729 tartrate were 0.500–500 ng/mL in plasma, 1.00–1,000 ng/mL in urine, and 0.100–10.0 μg/mL in feces, respectively.

The data acquisition and processing were performed by Analyst software (version 1.6.3, Applied Biosystems/Sciex, Foster, United States) and Watson LIMS software (version 7.5, Thermo Fisher Scientific, Waltham, MA, United States), respectively. The PK parameters were calculated with WinNonlin version 8.3 (Pharsight Corporation, Mountain View, California) using non-compartmental method. Other statistical analysis was performed by using SAS statistical package version 9.4 (SAS Research and Development Co., Ltd., Chapel Hill, NC, United States). The main PK parameters included maximum drug concentration in plasma (C_max_), the time to C_max_ (T_max_), terminal elimination half-life (t_1/2z_), area under the concentration-time curve from zero to the last measurable concentration (AUC_0-t_) and infinity (AUC_0-∞_), renal clearance (CLr) and the cumulative excretion rates of YZJ-4729 in urine (fe_u_%) and feces (fe_f_%). The relationship between dosage and PK parameters (AUC and C_max_) was analyzed using the power function model. The model was described as: $\text{PK}=\alpha \times {\text{dose}}^{\beta }$ (Hummel et al., 2009). Dose proportionality was confirmed when the 95% confidence interval (CI) of the β value contained the value of 1.000.

### 2.6 Metabolite profiling in human plasma, urine and feces

The metabolite profiling of YZJ-4729 in human biological matrices was conducted by using high performance liquid chromatography quadrupole time of flight mass spectrometry (HPLC-Q-TOF/MS) method. The HPLC-Q-TOF/MS analysis was performed on a Shimadzu LC-30AD HPLC system (Shimadzu, Kyoto, Japan), which was coupled with a X500B Q-TOF system (Applied Biosystems/Sciex, CA, United States). For the metabolite identification of YZJ-4729 tartrate in human plasma, urine and feces, samples were pooled according to the time point or interval. For each time point or interval, the samples from the subjects in the 1.5 mg dose cohort (except for the placebo group) were pooled in equal volume (100 μL). For the semi-quantitative profiling in circulation, the trapezoidal rule was used to yield the pooled plasma samples that had the concentration proportional to the AUC (Hamilton et al., 1981; Hop et al., 1998) for each subject. The plasma samples ranged from 0 h to 12 h post-dosing in the 1.5 mg dose cohort were used to prepare the AUC pools for each subject (except for the placebo group).

The strategy for metabolite profiling and identification was as follows. The first step was on-line data acquisition. It was performed in the full scan mode and depended on multiple mass defect filtering (MMDF) and dynamic background subtraction (DBS) techniques to capture all the probable metabolites (Zhang et al., 2017; Yang et al., 2023). The OS software (version 1.6.1, Applied Biosystems/Sciex, CA, United States) was used for the data acquisition. The second step was post-acquisition data processing. The MetabolitePilot™ 2.0.4 software (Applied Biosystems/Sciex, CA, United States) and its data-mining tools were employed to screen the probable metabolites of YZJ-4729. Finally, based on the accurate mass data, fragmentation pathways of the parent drug, and related drug biotransformation information, the metabolites were identified. The proportion of the parent drug and its metabolites in the AUC-pooled plasma samples represented their relative amounts in circulation. It was determined by the ratio of their peak area to the total peak area.

### 2.7 Safety and tolerability assessments

Safety and tolerability were assessed by measurements of adverse events (AEs), serious adverse events (SAEs), laboratory examination, ECG, oxygen saturation, end-tidal carbon dioxide, vital signs, physical examination, injection site reaction, addiction score, sleepiness score, and nausea/vomiting score. All AEs were recorded and coded by MedDRA (25.0 version). AEs were graded as follows: 1) mild: usually transient, no or minimal intervention indicated, and activities of daily living (ADL) not limited; 2) moderate: ADL limited, intervention indicated for relief, and no risk of significant or permanent harm; 3) severe: ADL disabled, and urgent intervention indicated. For the tolerability assessment, dose escalation would be stopped when one of the following scenarios occurred: 1) in one dose cohort, moderate or severe drug-related AEs occurred in half or more of the subjects; 2) in one dose cohort, 3 or more subjects reported severe drug-related AEs; 3) in one dose cohort, drug-related SAE was reported; 4) based on the clinical data and manifestation of the subjects, investigators confirmed dose escalation was unnecessary or inappropriate to be continued.

Based on the preclinical study results of YZJ-4729, common AEs of opioid analgesics and safety information from oliceridine, the following points should be noted during this clinical trial. 1) During the infusion, if severe dizziness, nausea, vomiting, constipation, rash, or itching occurs, the administration should be immediately discontinued. Symptomatic treatment should be provided, including antihistamines, antiemetics, glucocorticoids, and other supportive measures. 2) YZJ-4729 may cause respiratory depression. Close monitoring is needed. If respiratory depression happens, the administration should be immediately discontinued, and the subject should be provided with oxygen. If necessary, an artificial airway or mechanical ventilation should be established, and naloxone should be intravenously administered. 3) Opioid drugs may cause neurotoxicity. If excessive sedation, hallucinations, delirium, muscle spasms, seizures, or hyperalgesia occurs, the administration should be discontinued immediately and symptomatic treatment should be provided. 4) If anaphylactic shock occurs, appropriate rescue measures should be promptly provided, which including administering oxygen, intravenously injecting adrenaline, and monitoring vital signs such as blood pressure and respiration.

## 3 Results

### 3.1 Study population

A total of 54 subjects were enrolled in this study: 44 were dosed with YZJ-4729 tartrate injection and 10 were dosed with placebo across six dose levels. The safety analysis set (SS) included all the randomized subjects. The pharmacokinetics set (PKS) included all subjects who received YZJ-4729 tartrate injection. The baseline demographic characteristics of the study population are summarized in Table 1. Among the 54 subjects, gender distribution was balanced with 50% male subjects and 50% female subjects. The age of the subjects ranged from 20 to 43 years, and the body mass index was between 19.3 and 25.8 kg/m^2^.

**TABLE 1:** Baseline demographic data of the study population.

Characteristic	Dose cohort						
	0.2 mg (n = 4)	0.5 mg (n = 8)	1.5 mg (n = 8)	3 mg (n = 8)	4.5 mg (n = 8)	6 mg (n = 8)	Placebo (n = 10)
Sex, n							
Male/female	2/2	4/4	4/4	4/4	4/4	4/4	5/5
Age, years							
Mean (SD)	26.5 (4.43)	22.1 (4.12)	28.3 (6.41)	24.6 (4.03)	24.6 (4.00)	26.4 (3.34)	28.9 (6.40)
Min-Max	23–33	20–32	23–38	21–34	20–31	22–31	21–43
Height, cm							
Mean (SD)	160.63 (9.86)	167.00 (9.50)	162.50 (8.41)	162.19 (5.00)	163.31 (8.61)	160.75 (7.96)	162.70 (7.42)
Min-Max	147.5–170	155.5–184.5	154.5–175.5	155.5–168	149.5–178	150–175	153–179
Weight, kg							
Mean (SD)	60.70 (8.43)	58.56 (9.41)	62.35 (7.99)	57.45 (6.95)	60.01 (7.73)	59.91 (6.00)	58.52 (3.83)
Min-Max	49.7–67.4	48.6–78.7	53.8–78.1	46.7–68.1	45.5–72.0	50.5–67.1	53.1–64.3
BMI, kg/m^2^							
Mean (SD)	23.40 (0.70)	20.88 (1.11)	23.58 (2.10)	21.76 (1.59)	22.44 (1.51)	23.18 (1.58)	22.19 (1.94)
Min-Max	22.8–24.4	19.7–23.1	19.6–25.9	19.3–24.1	20.4–25.5	21.5–25.8	19.6–25.7

BMI, body mass index; SD, standard deviation; Max, maximum; Min minimum.

### 3.2 Pharmacokinetics

**FIGURE 2:**
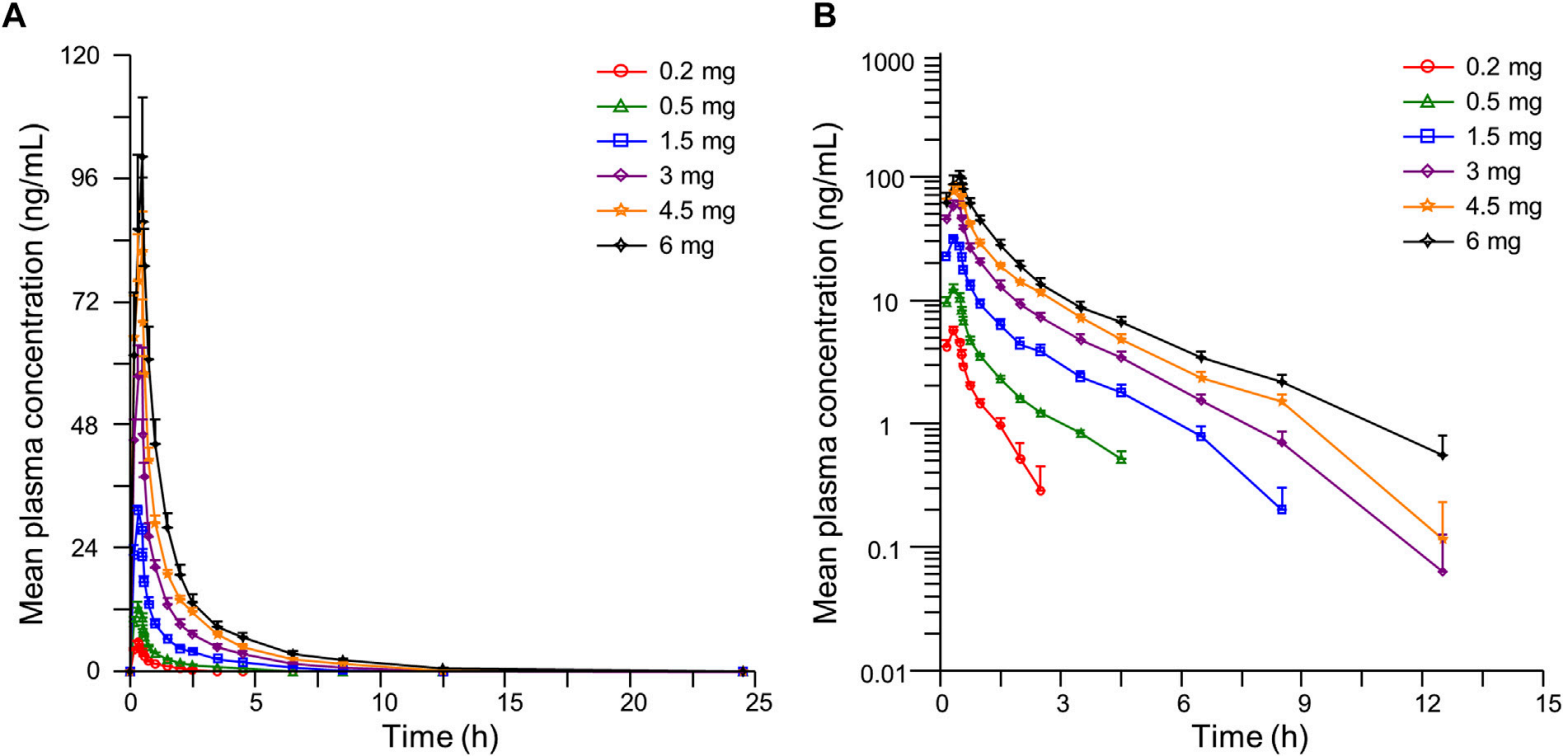
Mean plasma concentration-time curves of YZJ-4729 in healthy Chinese subjects after single intravenous administration. **(A)** linear scale. **(B)** semi-logarithmic scale. Bars represent SDs.

**FIGURE 3:**
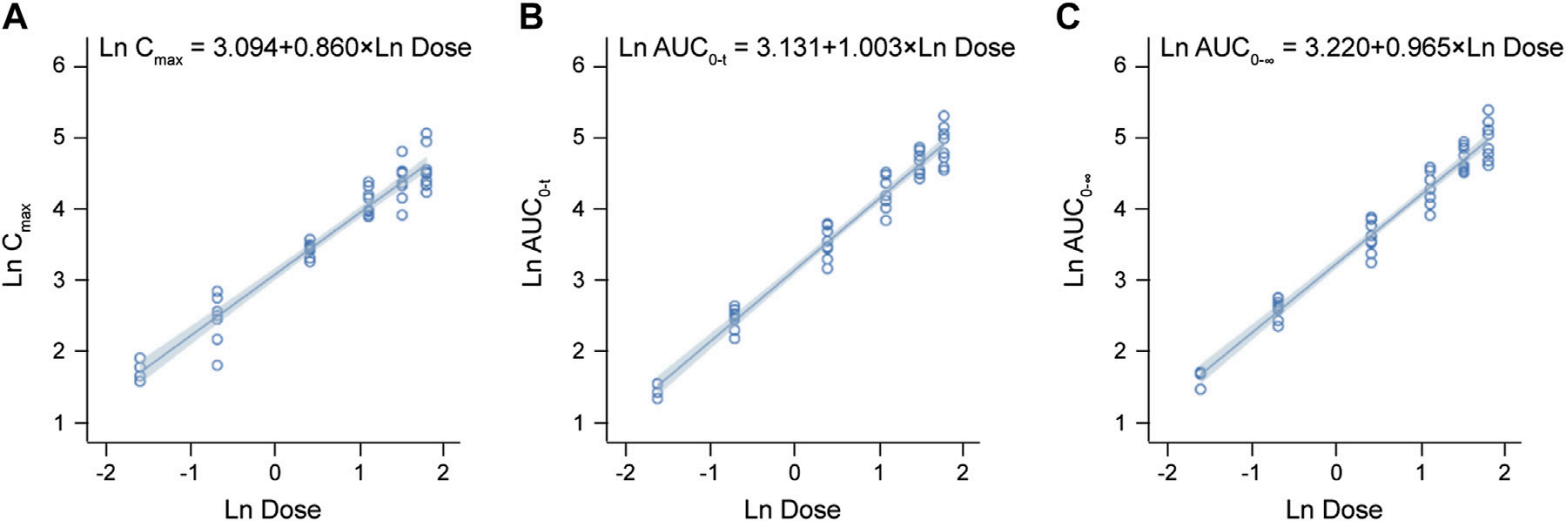
Relationships between PK parameters and dose of YZJ-4729 after single intravenous doses. **(A)** C_max_. **(B)** AUC_0-t_. **(C)** AUC_0-∞_.

The detailed pharmacokinetic parameters of YZJ-4729 are summarized in Table 2. The gender-specific analysis of the main PK parameters is shown in Table 3. The mean plasma concentration-time profiles of YZJ-4729 following single doses are presented in Figure 2. The relationships between PK parameters and dose of YZJ-4729 after single intravenous doses are shown in Figure 3. In the 0.2–3 mg dose cohorts, YZJ-4729 reached peak plasma concentrations at around 20 min after the start of the infusion. In the 4.5 mg and 6 mg dose cohorts, the C_max_ of the study drug was attained at the end of the 30-min infusion. Across all doses evaluated in the study, the mean terminal elimination half-life of YZJ-4729 ranged from 0.862 to 2.50 h. After single intravenous dose of 1.5 mg of YZJ-4729 tartrate, the cumulative excretion rate in urine was 0.274% over the time intervals of 0–72 h, and the renal clearance was 117 mL/h. However, because the drug concentration in feces samples was too low to be detected, the cumulative excretion rate in feces could not be evaluated. These results implied the study drug might be metabolized extensively in human body. Dose-related increases in YZJ-4729 exposure were observed across the entire dose range. The estimated β values and 95% confidence intervals for C_max_, AUC_0-t_, and AUC_0-∞_ calculated by the power model were 0.860 (0.795–0.926), 1.003 (0.948–1.059) and 0.965 (0.909–1.020), respectively. The 95% confidence intervals of β values for AUC included the value of 1, and the 95% confidence interval of β value for C_max_ was slightly below the value of 1.

**TABLE 2:** Main pharmacokinetic parameters of YZJ-4729 in healthy Chinese subjects after single intravenous doses.

Pharmacokinetic parameters	Dose cohort					
	0.2 mg (n = 4)	0.5 mg (n = 8)	1.5 mg (n = 8)	3 mg (n = 8)	4.5 mg (n = 8)	6 mg (n = 8)
C_max_, ng/mL	5.70 ± 0.82	12.25 ± 3.57	31.26 ± 3.08	61.95 ± 11.86	84.06 ± 22.08	101.30 ± 32.22
T_max_, h	0.334 (0.333–0.334)	0.334 (0.333–0.334)	0.334 (0.333–0.334)	0.335 (0.333–0.506)	0.501 (0.334–0.531)	0.497 (0.341–0.526)
AUC_0-t_, ng h/mL	4.36 ± 0.44	11.9 ± 1.7	34.9 ± 7.7	71.6 ± 16.3	106 ± 18	140 ± 39
AUC_0-∞_, ng h/mL	5.12 ± 0.54	13.5 ± 1.8	36.8 ± 8.1	73.7 ± 16.6	110 ± 19	144 ± 40
t_1/2z_, h	0.862 ± 0.331	1.81 ± 0.30	1.88 ± 0.43	2.00 ± 0.70	2.31 ± 0.70	2.50 ± 0.73
fe_u_, %	NA	NA	0.274 ± 0.203	NA	NA	NA
CLr, mL/h	NA	NA	117 ± 72	NA	NA	NA

C_max_, maximum drug concentration in plasma; T_max_, time to C_max_; AUC_0-t_, area under the concentration-time curve from zero to the last measurable concentration; AUC_0-∞_, area under the concentration-time curve from zero to infinity; t_1/2z_, terminal elimination half-life; CLr, renal clearance; fe_u_%, the cumulative excretion rate in urine; NA, not applicable. All values are expressed as mean ± SD, except for T_max_ values, which are expressed as median (range).

**TABLE 3:** Gender-specific analysis of main pharmacokinetic parameters of YZJ-4729 after single intravenous doses.

Pharmacokinetic parameters	Dose cohort											
	0.2 mg		0.5 mg		1.5 mg		3 mg		4.5 mg		6 mg	
	Male (n = 2)	Female (n = 2)	Male (n = 4)	Female (n = 4)	Male (n = 4)	Female (n = 4)	Male (n = 4)	Female (n = 4)	Male (n = 4)	Female (n = 4)	Male (n = 4)	Female (n = 4)
C_max_, ng/mL	6.34 ± 0.58	5.06 ± 0.26	12.66 ± 2.87	11.85 ± 4.58	30.85 ± 4.61	31.68 ± 0.71	58.85 ± 7.99	65.05 ± 15.45	67.70 ± 13.22	100.43 ± 15.76	79.93 ± 8.82	122.68 ± 33.55
T_max_, h	0.334 (0.333–0.334)	0.334 (0.333–0.334)	0.334 (0.333–0.334)	0.334 (0.333–0.334)	0.334 (0.333–0.334)	0.334 (0.333–0.334)	0.411 (0.333–0.506)	0.335 (0.333–0.486)	0.501 (0.334–0.531)	0.508 (0.334–0.521)	0.508 (0.484–0.526)	0.497 (0.341–0.517)
AUC_0-t_, ng h/mL	4.26 ± 0.62	4.46 ± 0.38	12.0 ± 1.4	11.8 ± 2.2	35.0 ± 11.0	34.9 ± 4.0	71.8 ± 15.5	71.5 ± 19.4	90.3 ± 4.3	121 ± 10	123 ± 27	158 ± 44
AUC_0-∞_, ng h/mL	4.89 ± 0.82	5.35 ± 0.04	13.6 ± 1.7	13.4 ± 2.2	37.0 ± 11.7	36.6 ± 4.0	73.3 ± 15.8	74.1 ± 19.7	93.7 ± 3.9	127 ± 10	125 ± 26	163 ± 46
t_1/2z_, h	0.675 ± 0.229	1.05 ± 0.37	1.77 ± 0.37	1.84 ± 0.25	1.61 ± 0.29	2.15 ± 0.40	1.90 ± 0.88	2.10 ± 0.58	2.14 ± 0.59	2.48 ± 0.76	2.18 ± 0.31	2.82 ± 0.95
fe_u_, %	NA	NA	NA	NA	0.374 ± 0.242	0.175 ± 0.108	NA	NA	NA	NA	NA	NA
CLr, mL/h	NA	NA	NA	NA	155 ± 74	79.0 ± 52.3	NA	NA	NA	NA	NA	NA

C_max_, maximum drug concentration in plasma; T_max_, time to C_max_; AUC_0-t_, area under the concentration-time curve from zero to the last measurable concentration; AUC_0-∞_, area under the concentration-time curve from zero to infinity; t_1/2z_, terminal elimination half-life; CLr, renal clearance; fe_u_%, the cumulative excretion rate in urine; NA, not applicable. All values are expressed as mean ± SD, except for T_max_ values, which are expressed as median (range).

### 3.3 Metabolite profiling in human plasma, urine and feces

**FIGURE 4:**
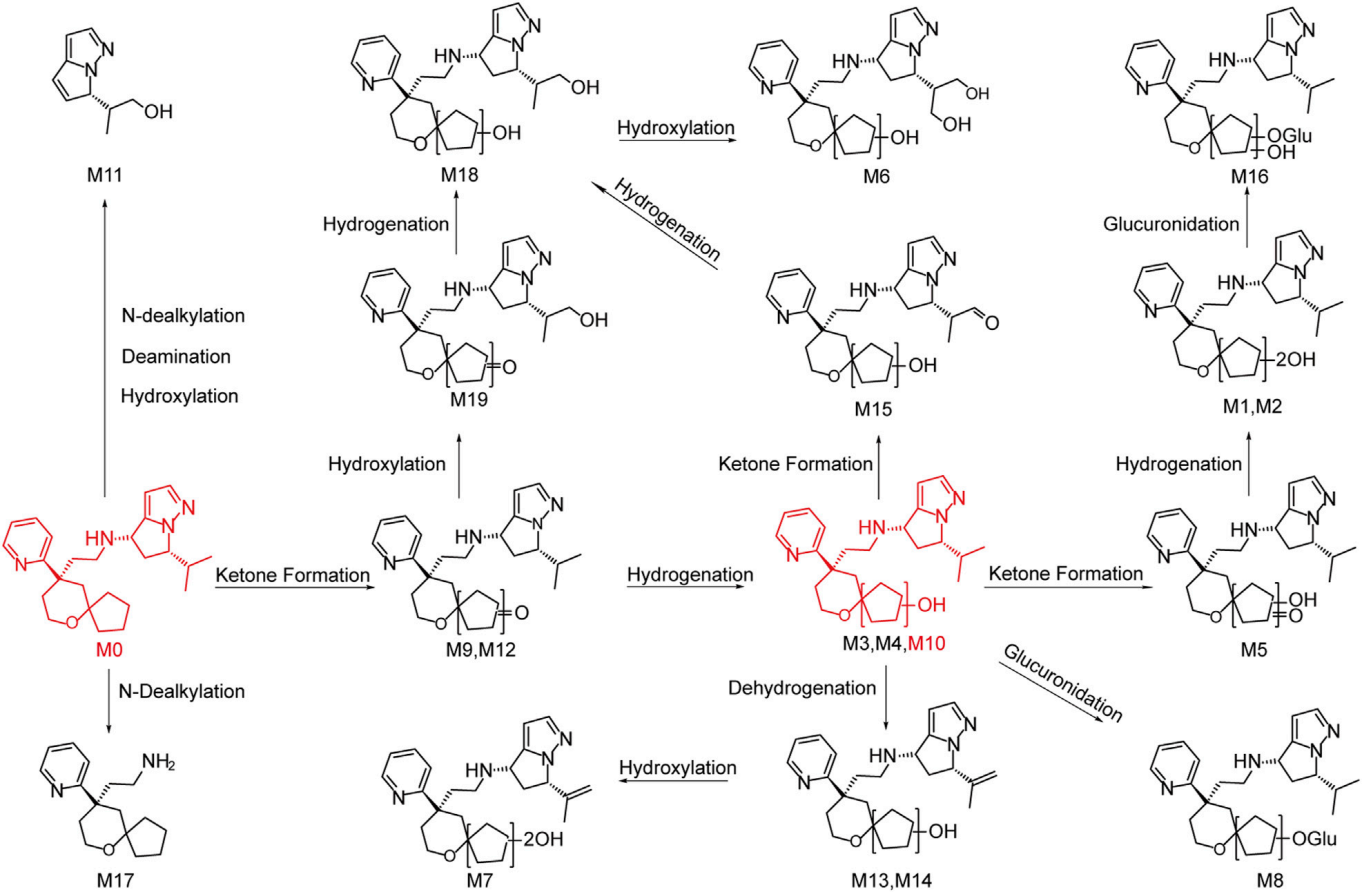
Chemical structures of the metabolites and proposed metabolic pathways of YZJ-4729.

YZJ-4729 and 19 metabolites were identified in human biological matrices with HPLC-Q-TOF/MS method. The characteristic metabolic pathways involved hydroxylation, ketone formation, N-dealkylation and glucuronide conjugation. As shown in Figure 1, to characterize the structures of metabolites clearly, the structure of YZJ-4729 was divided into 2 parts, Part A and Part B. The detailed information, structures of the metabolites and the proposed metabolic pathways are shown in Table 4 and Figure 4, respectively.

**TABLE 4:** Summary of YZJ-4729 and its metabolites *in vivo*.

Metabolite ID	Metabolite Description	Formula	m/z	Error (ppm)	Retention time (min)	Plasma	Urine	Feces	Relative contents in circulation^a^ (%)		
									Total	Male	Female
M0	Parent	C_25_H_36_N_4_O	409.2950	−2.8	30.46	+	-	-	56.69	61.52	51.87
M1	Di-Hydroxylation	C_25_H_36_N_4_O_3_	441.2870	2.1	10.51	+	+	+	0.84	-*	0.84
M2	Di-Hydroxylation	C_25_H_36_N_4_O_3_	441.2860	0.0	12.71	+	+	+	3.42	3.40	3.45
M3	Mono-Hydroxylation	C_25_H_36_N_4_O_2_	425.2921	2.3	13.48	+	+	-	1.14	-*	1.14
M4	Mono-Hydroxylation	C_25_H_36_N_4_O_2_	425.2906	−1.1	14.24	+	+	-	4.85	4.45	5.25
M5	Mono-Hydroxylation + Ketone Formation	C_25_H_34_N_4_O_3_	439.2711	1.6	14.78	+	+	-	-*	-*	-*
M6	Tri-Hydroxylation	C_25_H_36_N_4_O_4_	457.2815	1.3	14.83	+	+	+	1.00	-*	1.00
M7	Di-Hydroxylation + Dehydrogenation	C_25_H_34_N_4_O_3_	439.2709	1.2	16.13	+	+	-	1.15	1.29	1.11
M8	Mono-Hydroxylation + Glucuronidation	C_31_H_44_N_4_O_8_	601.3239	1.2	16.78	+	+	-	-*	-*	-*
M9	Ketone Formation	C_25_H_34_N_4_O_2_	423.2760	1.3	17.68	+	+	-	2.95	2.64	3.26
M10	Mono-Hydroxylation	C_25_H_36_N_4_O_2_	425.2901	−2.4	18.36	+	+	+	20.91	19.07	22.75
M11	N-dealkylation + Deamination + Hydroxylation	C_9_H_12_N_2_O	165.1021	−1.0	20.15	+	+	-	-*	-*	-*
M12	Ketone Formation	C_25_H_34_N_4_O_2_	423.2759	0.9	20.80	+	+	-	2.21	2.00	2.42
M13	Mono-Hydroxylation + Dehydrogenation	C_25_H_34_N_4_O_2_	423.2757	0.6	21.66	+	+	-	4.06	3.59	4.54
M14	Mono-Hydroxylation + Dehydrogenation	C_25_H_34_N_4_O_2_	423.2763	2.1	22.25	+	+	-	3.34	3.34	-*
M15	Mono-Hydroxylation + Ketone Formation	C_25_H_34_N_4_O_3_	439.2712	1.8	26.76	+	+	-	3.32	2.19	3.62
M16	Di-Hydroxylation + Glucuronidation	C_31_H_44_N_4_O_9_	617.3197	2.6	9.15	-	+	-	-	-	-
M17	N-Dealkylation	C_16_H_24_N_2_O	261.1966	1.6	12.89	-	+	-	-	-	-
M18	Di-Hydroxylation	C_25_H_36_N_4_O_3_	441.2859	−0.2	16.64	-	+	-	-	-	-
M19	Mono-Hydroxylation + Ketone Formation	C_25_H_34_N_4_O_3_	439.2705	0.3	19.28	-	+	-	-	-	-

^a^ Relative contents in circulation (%) = peak area of YZJ-4729 and each metabolite/total peak area in human plasma. +, detected; -, not detected; -*, not detected in AUC-pooled plasma sample.

#### 3.3.1 Fragmental pattern analysis of YZJ-4729

The MS/MS fragmentation of YZJ-4729 was investigated to facilitate the metabolite identification. The chromatographic and mass spectral properties of the parent drug would provide a structural template for interpreting the structures of the metabolites.

**FIGURE 5:**
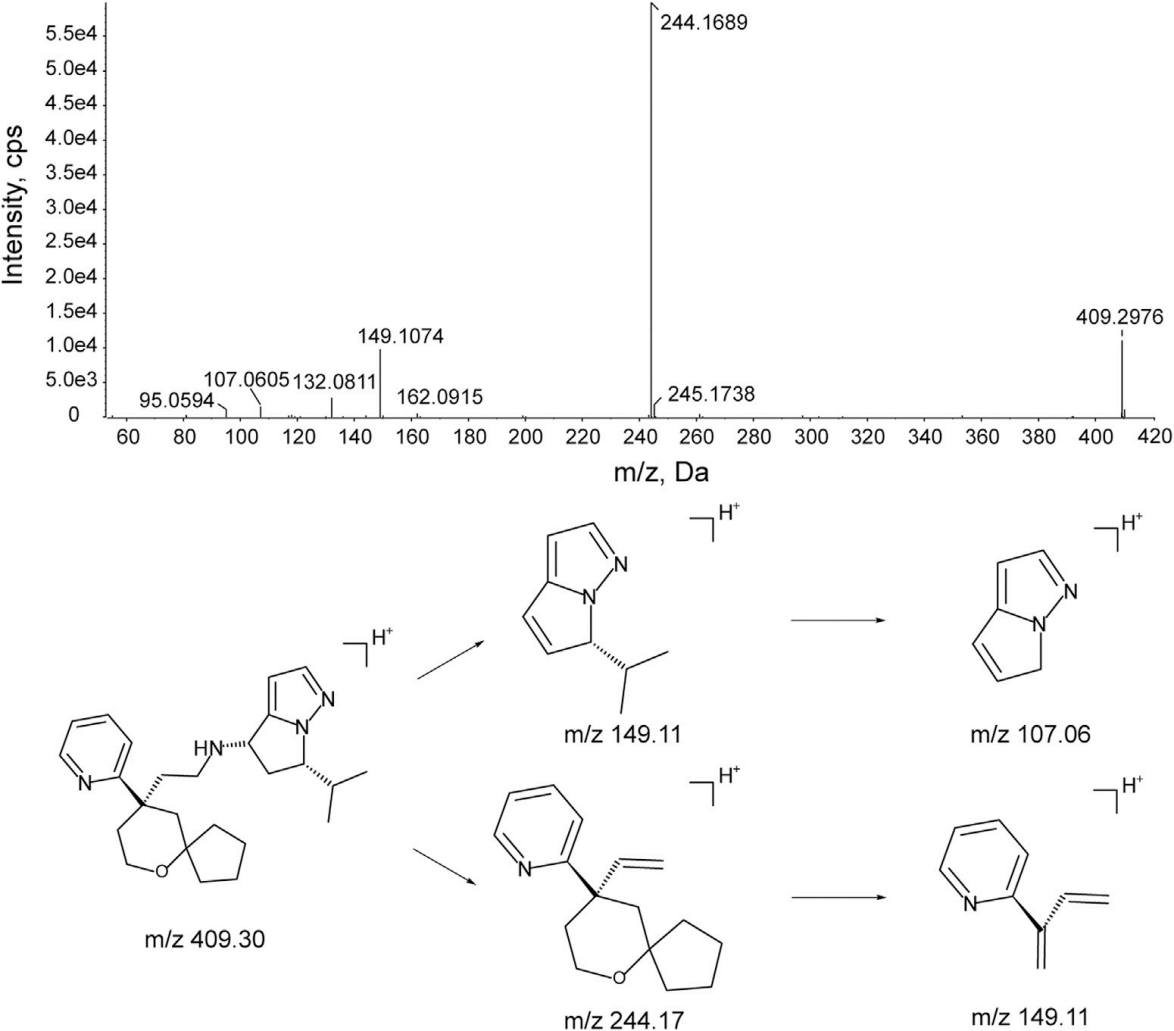
Accurate MS/MS spectrum of YZJ-4729 and its proposed fragmentation pathway.

The characteristic mass fragments of YZJ-4729 were investigated with its reference solution. YZJ-4729 was eluted at 30.46 min under the chromatographic conditions. The accurate mass measurement gave a protonated molecular ion ([M + H]^+^) at m/z 409.2976 with a theoretical elemental composition of C_25_H_36_N_4_O in the positive mode. The typical product ions at m/z 244.1689 (C_16_H_22_NO^+^), 149.1074 (C_9_H_13_N_2_^+^), 132.0811 (C_9_H_10_N^+^) and 107.0605 (C_6_H_7_N_2_^+^) were produced via the cleavage of the carbon-nitrogen bond and carbon-carbon bond. The proposed fragmentation pathways and MS/MS spectrum of YZJ-4729 are shown in Figure 5. By comparing the retention time (t_R_) and accurate mass spectra with those of the reference standard, the parent drug (M0) was identified in human plasma.

#### 3.3.2 Identification of metabolites from YZJ-4729

##### 3.3.2.1 Di-hydroxylation (metabolite M1, M2 and M18)

Metabolite M1, M2 and M18 (t_R_ = 10.51, 12.71 and 16.64 min, respectively) displayed the same [M + H]^+^ ions at around m/z 441.287 (C_25_H_36_N_4_O_3_) in the MS spectra, which were 32 Da higher than that of YZJ-4729. This indicated that they were the di-hydroxylated metabolites of the parent drug. M1 and M2 were observed in human plasma, urine and feces, and M18 was only observed in human urine. In the MS/MS spectra of M1 and M2, the product ion at m/z 149.11 was the same as that of YZJ-4729. Meanwhile, the product ion at m/z 276.16 was 32 Da higher than that of YZJ-4729 at m/z 244.17. Therefore, in terms of M1 and M2, di-hydroxylation might occur in the Part A. In the MS/MS spectrum of M18, the product ion at m/z 260.16 was 16 Da heavier than that of the parent drug at m/z 244.17, which indicated that mono-hydroxylation might occur in the Part A, and the other might occur in the Part B.

##### 3.3.2.2 Mono-hydroxylation (metabolite M3, M4 and M10)

Metabolite M3, M4 and M10 (t_R_ = 13.48, 14.24 and 18.36 min, respectively) showed the same [M + H]^+^ ions at around m/z 425.292 (C_25_H_36_N_4_O_2_), which were 16 Da more than that of YZJ-4729. M3 and M4 were detected in human plasma and urine, and M10 was detected in all human biological matrices. In the MS/MS spectra of M3, M4 and M10, the product ion at m/z 149.11 was consistent with that generated by YZJ-4729. Meanwhile, the product ion at m/z 260.16 was 16 Da higher than that of YZJ-4729 at m/z 244.17. Accordingly, M3, M4 and M10 were the mono-hydroxylated derivatives of YZJ-4729, and the mono-hydroxylation might occur in the Part A.

##### 3.3.2.3 Mono-hydroxylation and ketone formation (metabolite M5, M15 and M19)

Metabolite M5, M15 and M19 (t_R_ = 14.78, 26.76 and 19.28 min, respectively) were observed as [M + H]^+^ ions at around m/z 439.271 (C_25_H_34_N_4_O_3_), which were 2 Da less than that of the di-hydroxylated metabolites, indicating that they were the mono-hydroxylated and ketone formed metabolites of YZJ-4729. M5 and M15 were identified in human plasma and urine, and M19 was only identified in human urine. In the MS/MS spectrum of M5, the presence of the product ion at m/z 274.14 indicated that mono-hydroxylation and ketone formation occurred in the Part A. M15 produced an identical product ion at m/z 260.16, which was 16 Da more than that of YZJ-4729 at m/z 244.17, implying the mono-hydroxylation might occur in the Part A, and ketone formation might occur in the Part B. M19 produced a typical product ion at m/z 258.15, which was 14 Da more than that of YZJ-4729 at m/z 244.17, indicating the ketone formation might occur in the Part A, and mono-hydroxylation might occur in the Part B.

##### 3.3.2.4 Tri-hydroxylation (metabolite M6)

Metabolite M6 (t_R_ = 14.83 min), which was detected in human plasma, urine and feces, exhibited an [M + H]^+^ ion at m/z 457.281 (C_25_H_36_N_4_O_4_). The increase of 48 Da in molecular weight as compared to YZJ-4729 indicated that M6 was a tri-hydroxylated metabolite. Meanwhile, the product ion at m/z 260.16, which was 16 Da higher than that of the parent drug at m/z 244.17, indicated that one hydroxyl group was located in the Part A, and the other two hydroxyl groups were located in the Part B.

##### 3.3.2.5 Di-hydroxylation and dehydrogenation (metabolite M7)

Metabolite M7 (t_R_ = 16.13 min) gave an [M + H]^+^ ion at m/z 439.271 (C_25_H_34_N_4_O_3_), which was 30 Da heavier than that of YZJ-4729. It was detected in human plasma and urine. In its MS/MS spectrum, the product ion at m/z 147.09 was 2 Da less than that of YZJ-4729 at m/z 149.11, corresponding to the loss of a hydrogen molecule. Moreover, the product ion at m/z 276.16 was 32 Da more than that of YZJ-4729 at m/z 244.17, which was produced by the addition of two hydroxyl groups. Therefore, M7 was a di-hydroxylated and dehydrogenated metabolite of the study drug. The di-hydroxylation might occur in the Part A and the dehydrogenation might occur in the Part B.

##### 3.3.2.6 Mono-hydroxylation and glucuronidation (metabolite M8)

Metabolite M8 (t_R_ = 16.78 min) was found in human plasma and urine, which displayed an [M + H]^+^ ion at m/z 601.324 (C_31_H_44_N_4_O_8_). M8 shared the same product ions at m/z 260.16 and 149.11 with the mono-hydroxylated metabolites. Moreover, the diagnostic product ion at m/z 436.19 showed 176 Da (glucuronyl moiety) addition of the product ion at m/z 260.16, suggesting M8 was the glucuronide conjugate of the mono-hydroxylated metabolites.

##### 3.3.2.7 Ketone formation (metabolite M9 and M12)

Metabolite M9 and M12 (t_R_ = 17.68 and 20.80 min, respectively), with the same [M + H]^+^ ions at around m/z 423.276 (C_25_H_34_N_4_O_2_), were identified in human plasma and urine. In their MS/MS spectra, the product ion at m/z 258.15 was 14 Da heavier than that of YZJ-4729 at m/z 244.17, and 2 Da less than that of the mono-hydroxylated metabolites at m/z 260.16. This indicated M9 and M12 were the ketone formed metabolites of the parent drug, and the ketone formation might occur in the Part A.

##### 3.3.2.8 N-dealkylation, deamination and hydroxylation (metabolite M11)

Metabolite M11 (t_R_ = 20.15 min), which was detected in human plasma and urine, yielded an [M + H]^+^ ion at m/z 165.103 (C_9_H_12_N_2_O). Its product ion at m/z 147.09 was formed by the loss of water from the precursor ion. By comparing with the structure of the product ion of YZJ-4729 at m/z 149.11, M11 was proposed to be the N-dealkylated, deaminated and hydroxylated metabolite of the study drug.

##### 3.3.2.9 Mono-hydroxylation and dehydrogenation (metabolite M13 and M14)

Metabolite M13 and M14 (t_R_ = 21.66 and 22.25 min, respectively) had the same [M + H]^+^ ions at around m/z 423.276 (C_25_H_34_N_4_O_2_). They were both observed in human plasma and urine. In their MS/MS spectra, the presence of the product ion at m/z 260.16, which was 16 Da heavier than that of YZJ-4729 at m/z 244.17, implied that mono-hydroxylation might occur in the Part A. Meanwhile, the product ion at m/z 147.09 was 2 Da lower than that of YZJ-4729 at m/z 149.11, corresponding to the loss of a hydrogen molecule from the Part B of the parent drug. Accordingly, M13 and M14 were the mono-hydroxylated and dehydrogenated metabolite of YZJ-4729.

##### 3.3.2.10 Di-hydroxylation and glucuronidation (metabolite M16)

Metabolite M16 (t_R_ = 9.15 min) was only found in human urine. Its protonated molecular ion ([M + H]^+^) at m/z 617.319 (C_31_H_44_N_4_O_9_) was 176 Da more than that of the di-hydroxylated metabolites, which was assumed to result from the addition of a glucuronyl group. In its MS/MS spectrum, the product ion at m/z 276.16 was produced from m/z 452.15 by the loss of a glucuronyl group. The product ions at m/z 276.16 and 149.11 were consistent with those of M1 and M2. Hence, M16 was the glucuronide conjugate of the di-hydroxylated metabolites (M1 and M2).

##### 3.3.2.11 N-dealkylation (metabolite M17)

Metabolite M17 (t_R_ = 12.89 min), which was observed in human urine, showed an [M + H]^+^ ion at m/z 261.197 (C_16_H_24_N_2_O). It was 148 Da (C_9_H_12_N_2_) less than the parent drug. Its product ions at m/z 244.17 and 132.08 were same as those of the parent drug. Thus, M17 might be the N-dealkylated metabolite of YZJ-4729 and the cleavage of carbon-nitrogen bond might occur in Part B.

#### 3.3.3 Relative quantification of YZJ-4729 and metabolites in circulation

**FIGURE 6:**
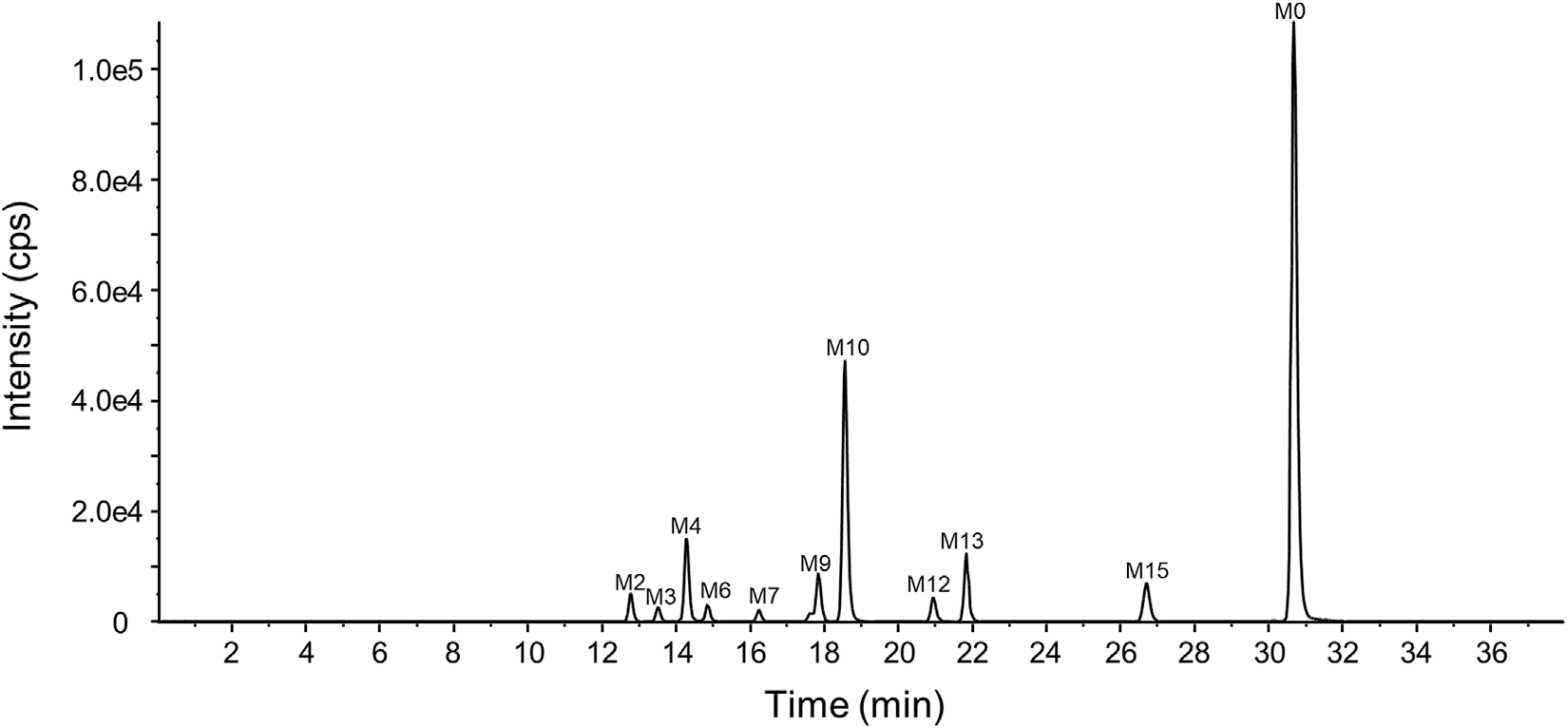
Representative chromatogram of the pooled plasma sample.

The representative chromatogram of the pooled plasma sample is shown in Figure 6. The relative contents of YZJ-4729 and each metabolite were displayed as the percentage of each extraction ion chromatograph peak area relative to the total peak area in the pooled plasma samples. As shown in Table 4, the unchanged YZJ-4729 represented the main proportion of total drug-related exposure (56.69% of the total peak area). Metabolite M10 was the most abundant circulating metabolite, which accounting for 20.91% of the total peak area. The other metabolites did not exceed 10% of total drug-related systemic exposure. There was no significant sex difference in drug metabolism. According to the FDA guidance, further PK study on M10 should be considered.

### 3.4 Safety and tolerability

In the subjects who received YZJ-4729 tartrate injection, 31 subjects reported 78 treatment-emergent adverse events (TEAEs). Among these TEAEs, 41 TEAEs were deemed as adverse drug reactions (ADRs) by the investigators. In the subjects who received placebo, 5 subjects reported 7 TEAEs, which were considered to be unrelated to the investigational drug. The details of TEAEs in subjects who received the study drug and placebo in the study are summarized in Supplementary Table S1. The measured values of oxygen saturation and end-tidal carbon dioxide at specific time points are displayed in Supplementary Tables S2, S3, respectively. All the TEAEs were mild except for 1 case of moderate oxygen desaturation, which was reported by 1 subject in the 6 mg dose cohort. During the electrocardiographic monitoring, the lowest recorded oxygen saturation of this subject was 77%. It was below 80%, and was assessed by the investigator as moderate. All events were resolved at the end of the study without any medical treatment. Three subjects received four cases of non-pharmacological treatments due to oxygen desaturation, which including chin lift and verbal reminders to breathe. These subjects then recovered spontaneously. Oxygen desaturation was observed in both male and female subjects. No SAEs or serious adverse reactions occurred in this study. The TEAEs reported by 2 or more subjects involved oxygen desaturation, sleepiness, nausea, dizziness, headache, fatigue and decreased heart rate. Because YZJ-4729 belonged to opioid analgesics, these TEAEs with high incidence were associated with its pharmacological effects. There was no off-target side effect in this study.

## 4 Discussion

This first-in-human clinical study investigated the pharmacokinetics, metabolite profiling, safety and tolerability of YZJ-4729 in healthy Chinese subjects following the single intravenous administration.

### 4.1 Safety and tolerability

The test results showed that YZJ-4729 tartrate was safe and well tolerated throughout the study. The TEAEs were generally mild and transient, and the overall incidences were not dose-dependent. The single doses of YZJ-4729 tartrate did not cause serious adverse events or discontinuations. The TEAEs with high incidence were related to the opioid pharmacology of the study drug.

In this study, the adverse events related to respiratory depression were resolved at the end of the study without any medical treatment. Generally, naloxone, an opioid receptor antagonist, was used in the treatment of opioid-overdose-induced respiratory depression (Van Drop et al., 2007). In our study, three subjects suffering oxygen desaturation received four cases of non-pharmacological treatment, including chin lift and verbal reminders to breathe. These subjects then recovered spontaneously. Other TEAEs were also resolved at the end of the study without any medical treatment. The overall data supported further studies to investigate a potentially superior safety profile of the biased, selective partial MOR agonist YZJ-4729.

### 4.2 Dose range design

The dose range used in this PK study was determined based on the preclinical safety, tolerability, and pharmacokinetic studies of YZJ-4729.

#### 4.2.1 Starting dose design

The starting dose was identified according to China National Medical Products Administration (NMPA) guidance for estimating the maximum recommended starting dose (MRSD) of drugs in the first clinical trial of healthy adult volunteers (China National Medical Products Administration, 2012).

##### 4.2.1.1 Based on no observed adverse effect levels (NOAELs)

Generally, MRSD was estimated on the basis of the NOAELs in multiple species. After the NOAELs in multiple species were achieved, they were converted to human equivalent doses (HEDs) based on the method of normalization to body surface area. The species that generated the lowest HED was deemed as the most sensitive one. Then, the MRSD was obtained from dividing the lowest HED by a safety factor. Usually, the safety factor was selected as 10. The safety factor was to assure that the first dose in humans would not cause adverse effects.

In the 14-day repeated dose toxicity study in SD rats and cynomolgus monkeys, the NOAELs were 5 and 6 mg/kg, respectively. The calculated HEDs were 1.01 and 2.43 mg/kg, respectively. In the safety pharmacology studies in rats, the NOAEL values were 1.5 mg/kg and 5 mg/kg, respectively, which achieved from the central nervous system (CNS) and respiratory system of SD rats. In the safety pharmacology studies in cynomolgus monkeys, the NOAEL value was 3 mg/kg, which achieved from the cardiovascular system. By using the data from the above safety pharmacology studies in rats and cynomolgus monkeys, the calculated HEDs were 0.30, 1.01 and 1.21 mg/kg, respectively. Above all, the central nervous system of SD rats was the most sensitive and the HED of 0.30 mg/kg was used to calculate the MRSD. When the safety factor was 10, and human body weight was assumed as 60 kg, the estimated MRSD was 1.82 mg.

##### 4.2.1.2 Based on systemic exposure under maximum tolerated doses (MTDs) and NOAELs

Another method to estimate the MRSD was based on the systemic exposure to YZJ-4729 in animals under the doses of MTDs and NOAELs. According to the drug plasma concentration at specific time points, the AUC, which represented the systemic exposure, was calculated. Then, combined with the PK parameters in humans predicted by the physiologically-based pharmacokinetic (PBPK) modeling, the HEDs were estimated. The lowest HED was used to calculate the MRSD. In this method, the lowest HED of 6.8 mg was obtained according to the systemic exposure under the NOAEL dose in the SD-rat CNS safety pharmacology study. It was used to calculate the MRSD. When the safety factor was 10, the MRSD was 0.68 mg.

##### 4.2.1.3 Based on pharmacologically-active exposure

In general, MRSD was the upper limit of the starting dose, and choosing a lower one would be more appropriate. However, excessively low starting dose might result in excessive dose escalation, and decrease the efficiency of the drug development. Therefore, pharmacologically-active exposure was taken into consideration in the starting dose design. Based on the preclinical efficacy models in rodents (hot plate and tail flick models) and their corresponding PK data, the population pharmacokinetic-pharmacodynamic (PopPK/PD) modeling was established to estimate the 20% effective concentration (EC_20_) in animals. Then through interspecies conversion, the drug plasma concentration in human that produced 20% of the maximum pharmacological activity was calculated. Combined with the human PK parameters predicted by PBPK modeling, the HED was estimated. Based on this method, the HED was 0.19 mg, which was lower than the MRSD of 0.68 mg.

##### 4.2.1.4 Based on similar drug

The dose range of oliceridine, the similar drug of YZJ-4729, in the first-in-human (FIH) study was 0.15–7 mg. In this dose range, oliceridine was safe and well-tolerated in healthy subjects.

In summary, after a comprehensive evaluation, the starting dose of YZJ-4729 was set as 0.2 mg in this study.

#### 4.2.2 Maximum dose design

According to the results of single-dose toxicity study in animals, after intravenous administration of YZJ-4729 in SD rats and cynomolgus monkeys, the MTD was 20 and 25 mg/kg, respectively. When calculated with 1/5 of the MTD in animals, the MTD in human was 240 and 300 mg, respectively. When using the systemic exposure under the NOAEL dose in the SD-rat CNS safety pharmacology study to estimate the HED, it was 6.8 mg. Meanwhile, the maximum dose of oliceridine was set as 7 mg. After a comprehensive evaluation, the maximum dose of YZJ-4729 was set as 6 mg in this study.

### 4.3 Pharmacokinetics

After the intravenous administration, YZJ-4729 exhibited the mean terminal elimination half-life of 0.862–2.50 h, which was similar to current opioid analgesics, such as morphine and oliceridine (Soergel et al., 2014; Lugo and Kern, 2002). The t_1/2z_ calculated from the initial dose (0.2 mg) was lower than other dose cohorts. This might be due to the low initial dose design and the analytical limitations at the lower end of the concentration range. The elimination profiles suggested that similar administration paradigms (bolus, continuous infusion, or patient-controlled) could be applied for the study drug (Soergel et al., 2014). Following single ascending dose administrations ranged from 0.2 mg to 6 mg, C_max_ and AUC values for YZJ-4729 increased with the dose. The 95% confidence interval of β value for C_max_ was slightly below the value of 1. This result might be related to the number of the subjects in this study. The number of the subjects in each dose cohort was relatively low and had an apparent impact on the strength of the dose-proportionality assessment. These PK results provided the foundation for establishing a safe and effective therapeutic dose schedule in clinical practice.

### 4.4 Metabolite profiling

YZJ-4729 was excreted little in human urine and feces, which implied that the study drug might be metabolized extensively in human body. According to the metabolite profiling and identification results, the parent drug was only detected in circulation, and there were multiple metabolites in human excreta. These results demonstrated that YZJ-4729 experienced extensive metabolism under the action of drug metabolizing enzymes. A total of 19 metabolites were observed in human plasma, urine and feces. The characteristic metabolic pathways involved hydroxylation, ketone formation, N-dealkylation and glucuronide conjugation. The metabolites, which were produced by these metabolic pathways, enhanced the water solubility of the study drug and facilitated its excretion. The activity of the metabolites on the opioid receptors would be investigated in the subsequent studies. There was no significant sex difference in drug metabolism.

According to the results of the *in vitro* metabolite profiling and identification studies, the major metabolic pathways of YZJ-4729 in the liver microsomes and hepatocytes from mice, SD rats, dogs, monkeys, and human included hydroxylation and N-dealkylation. The metabolites detected in human liver microsomes and hepatocytes were also detectable in at least one species of the above-mentioned animals. According to the results of metabolite profiling and identification studies in rats and cynomolgus monkeys, the most circulating component in plasma was the parent drug, which accounted for approximately 70% and 60% of total drug-related systemic exposure, respectively. 8 and 9 circulating metabolites were identified in rats and monkeys, respectively. Compared with the metabolites detected in human, the metabolic pathways unique to human (M6-M8 and M13-M16) included glucuronidation and hydroxylation, dehydrogenation and ketone formation in the Part B of the study drug. However, each of them accounted for less than 5% of total drug-related systemic exposure, which meant these metabolites might not raise a safety concern.

Metabolite M10, the mono-hydroxylated metabolite of the study drug, was the most abundant circulating metabolite. It represented over 10% of total drug-related systemic exposure. The safety testing of drug metabolites guidance for industry of FDA emphasizes human metabolites that present at greater than 10 percent of total drug-related exposure can raise a safety concern. Therefore, further PK and safety evaluation of M10 was necessary.

## 5 Conclusion

In conclusion, the pharmacokinetics, metabolite profiling, safety and tolerability of YZJ-4729 was evaluated in healthy Chinese subjects following the single intravenous administration. The clinical study results laid a foundation for the further clinical studies of YZJ-4729 in patients.

## Data Availability

The original contributions presented in the study are included in the article/Supplementary Material, further inquiries can be directed to the corresponding author.
