# Supplementary material for: Pharmacokinetics, metabolite profiling, safety and tolerability of YZJ-4729 tartrate, a novel G protein-biased μ-opioid receptor agonist, in healthy Chinese subjects
Source: Front Pharmacol. 2024 Jan 9;14:1295319. doi: 10.3389/fphar.2023.1295319 (PMC10803517; doi:10.3389/fphar.2023.1295319)
Supplement: Supplementary file 1 [file Table1.DOCX]

**Supplementary Table 1 Number of adverse events and percentage of healthy subjects in the study.**

| Adverse events | Dose cohort | | | | | | | |
| --- | --- | --- | --- | --- | --- | --- | --- | --- |
|  | 0.2 mg (n=4) | 0.5 mg (n=8) | 1.5 mg (n=8) | 3 mg (n=8) | 4.5 mg (n=8) | 6 mg (n=8) | Placebo (n=10) | Total (n=54) |
|  | n (%) | n (%) | n (%) | n (%) | n (%) | n (%) | n (%) | n (%) |
| Positive bacteria culture | 1(25.0) | 1(12.5) | 2(25.0) | 3(37.5) | 2(25.0) | 1(12.5) | 1(10.0) | 11(20.4) |
| Decreased oxygen saturation | 0(0) | 1(12.5) | 0(0) | 1(12.5) | 3(37.5) | 3(37.5) | 0(0) | 8(14.8) |
| Positive urinary white blood cells | 0(0) | 0(0) | 1(12.5) | 1(12.5) | 1(12.5) | 1(12.5) | 0(0) | 4(7.4) |
| Positive urinary red blood cells | 0(0) | 0(0) | 1(12.5) | 0(0) | 0(0) | 1(12.5) | 1(10.0) | 3(5.6) |
| Increased serum uric acid | 0(0) | 1(12.5) | 2(25.0) | 0(0) | 1(12.5) | 1(12.5) | 0(0) | 5(9.3) |
| Urinary sediment detected | 0(0) | 0(0) | 1(12.5) | 0(0) | 0(0) | 0(0) | 0(0) | 1(1.9) |
| Urinary protein detected | 0(0) | 0(0) | 0(0) | 2(25.0) | 1(12.5) | 0(0) | 0(0) | 3(5.6) |
| Positive urine occult blood | 0(0) | 0(0) | 0(0) | 0(0) | 0(0) | 1(12.5) | 1(10.0) | 2(3.7) |
| Decreased heart rate | 0(0) | 0(0) | 2(25.0) | 0(0) | 0(0) | 0(0) | 0(0) | 2(3.7) |
| Increased blood triglyceride | 0(0) | 0(0) | 1(12.5) | 0(0) | 0(0) | 1(12.5) | 0(0) | 2(3.7) |
| Increased bile acids | 0(0) | 0(0) | 0(0) | 0(0) | 1(12.5) | 0(0) | 1(10.0) | 2(3.7) |
| Positive urinary leukocyte esterase | 0(0) | 0(0) | 0(0) | 0(0) | 0(0) | 0(0) | 1(10.0) | 1(1.9) |
| Decreased blood fibrinogen | 0(0) | 0(0) | 2(25.0) | 0(0) | 0(0) | 0(0) | 0(0) | 2(3.7) |
| Decreased blood pressure | 0(0) | 0(0) | 0(0) | 0(0) | 0(0) | 1(12.5) | 0(0) | 1(1.9) |
| Increased diastolic blood pressure | 0(0) | 0(0) | 1(12.5) | 0(0) | 0(0) | 0(0) | 0(0) | 1(1.9) |
| Increased serum creatine phosphokinase | 0(0) | 0(0) | 0(0) | 0(0) | 1(12.5) | 0(0) | 0(0) | 1(1.9) |
| Decreased serum magnesium | 0(0) | 0(0) | 0(0) | 0(0) | 0(0) | 0(0) | 1(10.0) | 1(1.9) |
| Decreased blood glucose | 0(0) | 0(0) | 0(0) | 0(0) | 1(12.5) | 0(0) | 0(0) | 1(1.9) |
| Increased platelet count | 0(0) | 0(0) | 0(0) | 0(0) | 0(0) | 0(0) | 0(0) | 1(1.9) |
| Sleepiness | 0(0) | 1(12.5) | 1(12.5) | 2(25.0) | 3(37.5) | 1(12.5) | 0(0) | 9(16.7) |
| Dizziness | 0(0) | 0(0) | 0(0) | 0(0) | 2(25.0) | 1(12.5) | 0(0) | 3(5.6) |
| Headache | 0(0) | 0(0) | 0(0) | 0(0) | 0(0) | 2(25.0) | 0(0) | 2(3.7) |
| Hypoaesthesia | 0(0) | 0(0) | 0(0) | 0(0) | 0(0) | 1(12.5) | 0(0) | 1(1.9) |
| Vertigo | 0(0) | 0(0) | 0(0) | 0(0) | 1(12.5) | 0(0) | 0(0) | 1(1.9) |
| Abnormal sensation | 0(0) | 0(0) | 1(12.5) | 0(0) | 0(0) | 0(0) | 0(0) | 1(1.9) |
| Nausea | 1(12.5) | 0(0) | 1(12.5) | 0(0) | 2(25.0) | 3(37.5) | 0(0) | 7(13.0) |
| Dry mouth | 0(0) | 0(0) | 1(12.5) | 0(0) | 0(0) | 0(0) | 0(0) | 1(1.9) |
| Vomiting | 0(0) | 0(0) | 0(0) | 0(0) | 0(0) | 1(12.5) | 0(0) | 1(1.9) |
| Fatigue | 0(0) | 0(0) | 1(12.5) | 1(12.5) | 0(0) | 0(0) | 0(0) | 2(3.7) |
| Fever | 0(0) | 1(12.5) | 0(0) | 0(0) | 0(0) | 0(0) | 0(0) | 1(1.9) |
| Chest discomfort | 0(0) | 0(0) | 0(0) | 0(0) | 1(12.5) | 0(0) | 0(0) | 1(1.9) |
| Sinus bradycardia | 0(0) | 0(0) | 1(12.5) | 0(0) | 0(0) | 0(0) | 0(0) | 1(1.9) |
| Tinnitus | 0(0) | 0(0) | 0(0) | 1(12.5) | 0(0) | 0(0) | 0(0) | 1(1.9) |
| Insomnia | 0(0) | 1(12.5) | 0(0) | 0(0) | 0(0) | 0(0) | 0(0) | 1(1.9) |

n (%) = number and percentage of adverse events in each dose cohort.

**Supplementary Table 2 Measured oxygen saturation (%) at specific time points.**

| Time point | Dose cohort | | | | | | |
| --- | --- | --- | --- | --- | --- | --- | --- |
|  | 0.2 mg (n=4) | 0.5 mg (n=8) | 1.5 mg (n=8) | 3 mg (n=8) | 4.5 mg (n=8) | 6 mg (n=8) | Placebo (n=10) |
| Baseline | 98.5 (1.0) | 98.8 (0.9) | 98.5 (0.9) | 98.5 (0.8) | 98.3 (0.7) | 99.3 (0.7) | 98.4 (1.1) |
| 30 min after the start of infusion (just at the end of the infusion) | 98.5 (1.0) | 98.3 (0.7) | 97.4 (1.1) | 97.0 (1.2) | 96.5 (0.9) | 97.4 (1.6) | 98.3 (1.0) |
| 15 min post-doing | 97.8 (1.0) | 97.8 (0.7) | 97.3 (0.7) | 97.0 (1.1) | 97.5 (1.2) | 97.3 (1.5) | 98.1 (0.9) |
| 30 min post-doing | 98.5 (1.0) | 98.4 (0.9) | 97.8 (0.9) | 97.6 (1.1) | 97.1 (0.8) | 97.9 (1.3) | 98.1 (1.0) |
| 1 h post-doing | 98.3 (0.5) | 98.0 (0.8) | 97.9 (1.0) | 97.9 (1.0) | 97.9 (0.8) | 98.3 (0.7) | 98.1 (0.7) |
| 2 h post-doing | 99.3 (1.0) | 97.8 (0.7) | 97.9 (1.0) | 98.3 (1.0) | 98.4 (0.7) | 98.6 (0.7) | 98.4 (1.1) |
| 4 h post-doing | 97.5 (1.0) | 98.5 (0.9) | 97.6 (1.4) | 98.1 (0.8) | 98.0 (1.2) | 97.6 (0.7) | 98.3 (0.8) |
| 6 h post-doing | 98.0 (1.4) | 97.6 (1.1) | 98.9 (0.6) | 98.8 (1.0) | 98.5 (0.8) | 98.5 (0.9) | 98.3 (1.1) |
| 12 h post-doing | 98.0 (0.8) | 98.9 (0.8) | 98.3 (1.3) | 98.1 (1.5) | 98.9 (0.8) | 98.5 (0.9) | 98.5 (1.1) |
| 24 h post-doing | 98.5 (0.6) | 98.0 (1.1) | 98.3 (1.0) | 98.5 (0.8) | 98.5 (1.3) | 98.1 (1.0) | 98.5 (1.1) |
| 48 h post-doing | 97.3 (2.1) | 98.9 (0.8) | 99.1 (0.8) | 99.0 (0.5) | 98.0 (0.9) | 99.1 (0.8) | 98.6 (0.8) |
| Discharge examination | 98.8 (1.0) | 98.3 (1.0) | 99.3 (0.5) | 99.4 (0.7) | 98.4 (1.2) | 98.9 (1.0) | 98.9 (0.9) |

All values are expressed as mean (SD).

**Supplementary Table 3 Measured end-tidal carbon dioxide (mmHg) at specific time points.**

| Time point | Dose cohort | | | | | | |
| --- | --- | --- | --- | --- | --- | --- | --- |
|  | 0.2 mg (n=4) | 0.5 mg (n=8) | 1.5 mg (n=8) | 3 mg (n=8) | 4.5 mg (n=8) | 6 mg (n=8) | Placebo (n=10) |
| Baseline | 40.8 (0.96) | 36.8 (3.45) | 39.9 (3.27) | 40.5 (3.70) | 41.5 (4.99) | 39.5 (7.87) | 39.1 (4.58) |
| 30 min after the start of infusion (just at the end of the infusion) | 42.5 (1.73) | 40.3 (3.28) | 42.8 (3.62) | 46.6 (2.72) | 46.4 (6.00) | 47.6 (4.03) | 40.0 (2.11） |
| 15 min post-doing | 42.5 (1.91) | 40.0 (2.56) | 40.1 (5.19) | 45.4 (2.67) | 45.4 (4.21) | 44.8 (4.92) | 39.3 (2.75) |
| 30 min post-doing | 41.8 (2.63) | 39.3 (2.43) | 41.3 (2.31) | 43.1 (3.14) | 43.9 (4.88) | 44.4 (4.63) | 39.9 (3.41) |
| 1 h post-doing | 41.3 (2.50) | 39.3 (2.82) | 40.0 (3.70) | 43.1 (1.55) | 44.0 (3.63) | 42.9 (5.82) | 40.3 (3.43） |
| 1.5 h post-doing | 40.5 (1.29) | 39.9 (2.95) | 41.4 (2.39) | 42.9 (2.64) | 42.4 (5.13) | 41.9 (6.51) | 40.1 (3.48) |
| 2 h post-doing | 40.0 (3.56) | 39.4 (2.56) | 41.0 (2.56) | 43.1 (2.42) | 42.4 (3.89) | 41.6 (7.44) | 39.9 (3.45) |
| 3 h post-doing | 42.8 (1.26) | 37.6 (2.77) | 38.8 (4.27) | 42.8 (2.92) | 42.5 (3.51) | 40.8 (6.76) | 39.7 (3.43) |

All values are expressed as mean (SD).
